# Molecular interaction of metastasis suppressor genes and tumor microenvironment in breast cancer

**DOI:** 10.37349/etat.2023.00173

**Published:** 2023-10-11

**Authors:** Sathammai Sathappa Supuramanian, Sid Dsa, Sitaram Harihar

**Affiliations:** University of Kansas Medical Center, USA; Department of Genetic Engineering, SRM Institute of Science and Technology, Kattankulathur 603203, Tamil Nadu, India

**Keywords:** Tumor microenvironment, metastasis suppressor genes, breast cancer, breast cancer metastasis suppressor 1

## Abstract

Breast cancer (BC) is a leading cause of cancer-related deaths in women worldwide where the process of metastasis is a major contributor to the mortality associated with this disease. Metastasis suppressor genes are a group of genes that play a crucial role in preventing or inhibiting the spread of cancer cells. They suppress the metastasis process by inhibiting colonization and by inducing dormancy. These genes function by regulating various cellular processes in the tumor microenvironment (TME), such as cell adhesion, invasion, migration, and angiogenesis. Dysregulation of metastasis suppressor genes can lead to the acquisition of an invasive and metastatic phenotype and lead to poor prognostic outcomes. The components of the TME generally play a necessary in the metastasis progression of tumor cells. This review has identified and elaborated on the role of a few metastatic suppressors associated with the TME that have been shown to inhibit metastasis in BC by different mechanisms, such as blocking certain cell signaling molecules involved in cancer cell migration, invasion, enhancing immune surveillance of cancer cells, and promoting the formation of a protective extracellular matrix (ECM). Understanding the interaction of metastatic suppressor genes and the components of TME has important implications for the development of novel therapeutic strategies to target the metastatic cascade. Targeting these genes or their downstream signaling pathways offers a promising approach to inhibiting the spread of cancer cells and improves patient outcomes.

## Introduction

Breast cancer (BC) is a diverse disease that is the most prevalent cancer in the world, particularly among women [1]. BC causes more women to lose disability-adjusted life years (DALYs) than other cancers. The World Health Organization (WHO) estimates that 2.3 million women receive a BC diagnosis each year. BC starts in the lining cells (epithelium) of the ducts (85%) or lobules (15%) of the glandular tissue of the breast [2]. Estrogen exposure can alter the genetic alterations and DNA damage that leads to BC. Classification of BC depends on the presence or absence of estrogen receptor alpha (ERα), progesterone receptor (PR), and human epidermal growth factor receptor 2 (HER2). Invasive or infiltrating pre-cancer, which is less common and more severe, spreads into the surrounding breast tissue after starting in a milk duct. The *in situ* pre-cancer, which begins in a milk duct but does not extend to the rest of the breast tissue, is distinct from the invasive one. One in eight women (12.4%) may get invasive BC in their lifetime. Frequent screening for BC, primarily via mammography, can significantly decrease the chance of death from the disease [3]. Some of the risk factors, both changeable and immutable, include age, family history, mutations, alcohol consumption, smoking, and more [4]. Furthermore, a significant percentage of those who are diagnosed with BC disclose having a first-degree relative who has the disease [5]. BC susceptibility gene 1 (*BRCA1*) and *BRCA2* are two significant genes with high penetrance found on chromosomes 17 and 13, respectively. They primarily raise the possibility of developing BC [6]. An assortment of breast tumors that are ER-negative, PR-negative, and HER2-negative collectively fall under the term “triple-negative BC (TNBC)”. TNBC accounts for over 80% of BCs with BRCA1 germline mutations, while BRCA1 or BRCA2 germline mutations account for 11–16% of all TNBCs. TNBC frequently has a worse prognosis and is physiologically more aggressive [7, 8]. Some of the treatment strategies include surgery, radiotherapy, chemotherapy, and hormone therapy [4].

The biological mechanism known as the metastatic cascade allows for the spread of cancerous cells from their original site to a secondary site and the emergence of new cancer cells [9]. In contrast to other organs like the liver and brain, BC has been observed to spread more frequently to the bone and lungs [10]. It is well-recognized that circulating tumor cells are crucial to the metastasis of carcinomas. Because a favorable microenvironment is vital for the growth and progression of malignant tumors, it is necessary for the proliferation of metastatic tumor cells [11]. The possibility exists that tumor cells may manufacture substances to prepare the “soil” prior to metastasis in order to establish a “pre-metastatic niche” that would support future metastatic locales [10, 12]. The evidence is mounting in favor of the hypothesis that strategies emphasizing the interactions between the tumor and TME may pave the way for a new generation of therapies. Numerous pre-clinical or clinical studies have demonstrated that targeting tumor microenvironment (TME) with directed therapy reduces tumor growth, metastasis, and chemoresistance [13, 14]. Interaction between the tumor cell and its microenvironment is necessary to establish metastasis; therefore, cutting off this interaction should lessen the likelihood of metastasis.

A protein known as a metastasis suppressor slows or stops metastases (secondary tumors) from growing and spreading throughout the body of a cancerous organism [15]. Metastasis suppressors frequently appear to selectively regulate how cells react to environmental signals by modifying signaling cascades that regulate downstream gene expression [16, 17]. Nevertheless, metastasis suppressors exist in both cells and the extracellular milieu; they each have a unique method of action and control a different stage of the metastatic cascade [16]. This review’s primary focus is metastasis suppressor genes related to BC and their function in regulating the TME. Understanding the interaction between the components of the TME and metastasis suppressor genes in order to inhibit the metastasis of BC may pave the way for potential therapeutic opportunities.

## TME in BC

A tumor is a heterogeneous assemblage of invading and resident host cells, secreted chemicals, and extracellular matrix (ECM) rather than just a collection of cancer cells [18]. Tumor growth and progression are influenced by two distinct pathways, namely genetic and epigenetic alterations in the tumor cells and the reorganization of the TME elements through reciprocal and dynamic interaction [19]. Through intricate signaling networks, tumor cells, the driving force being the TME, regulate cellular and no-cellular functions to take advantage of non-malignant cells for their interests [19, 20].

**Figure 1:**
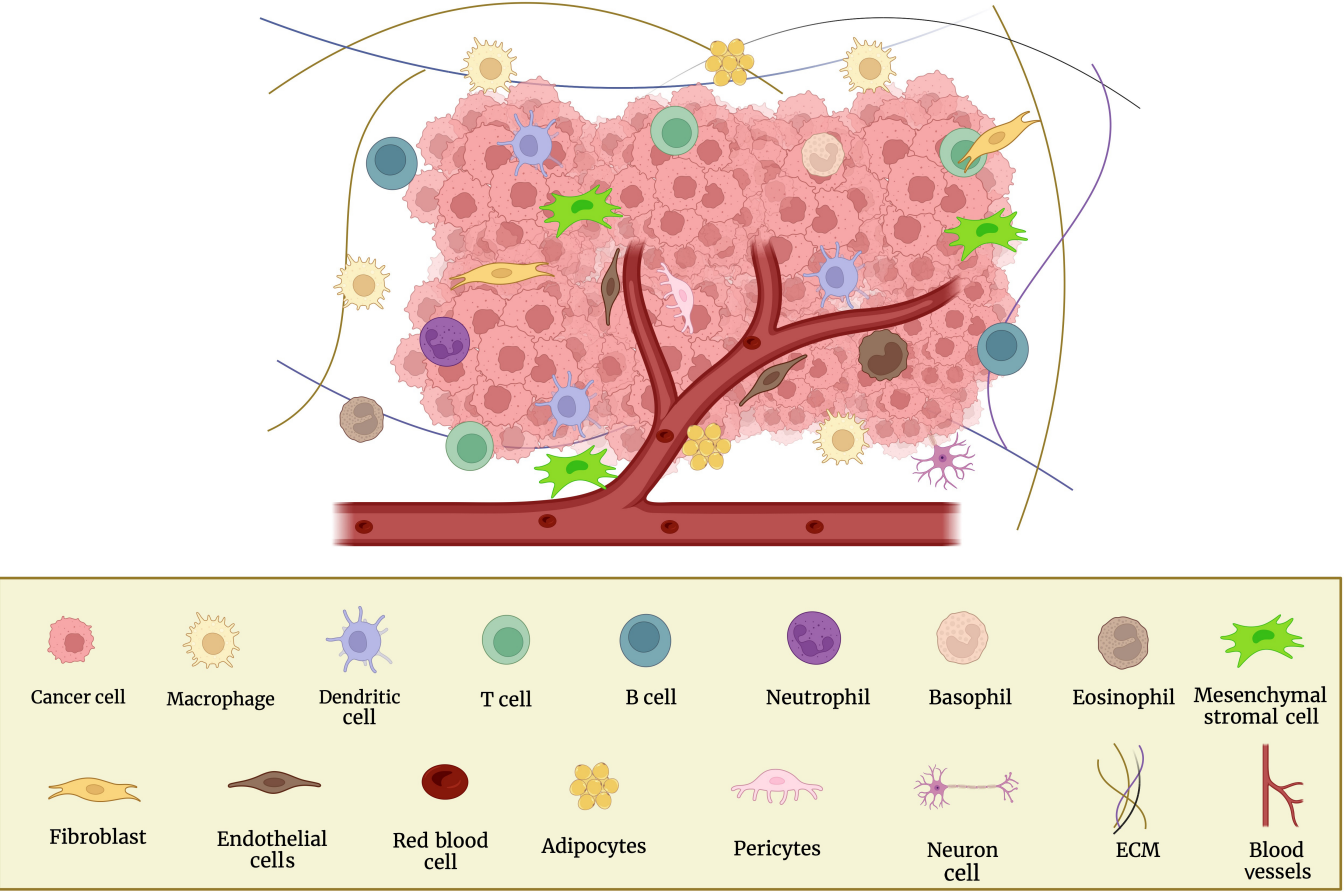
Cellular components of the TME and surrounding BC cells. This figure was created with BioRender

The stages of the metastatic cascade depend on interactions between the microenvironment and cancer cells. Immune cells and their mediators promote metastatic formation in this regional milieu and distant organs. Blood vessels, fibroblasts, ECM, signaling molecules, immune cells, non-neoplastic cells, tumor cells, and more make up the TME (Figure 1), which promotes the growth and spread of cancer [19, 21]. Using a variety of markers, the cellular segmentation of tumors and their associated TMEs allowed the detection of tumor-associated macrophages (TAMs), cancer-associated fibroblasts (CAFs), T and B lymphocytes, natural killer (NK) cells, and cancer stem cells (CSCs) [22]. The microenvironments in which tumors grow, invade, and metastasize are intricate and dynamic. In order to target them and use them as a potential therapeutic target, it is crucial to understand the involvement of the TME in BC.

In the microenvironment of solid tumors, fibroblasts are one of the most prevalent cell types. Breast, pancreatic, colon, and prostate carcinomas are among those where fibroblasts are most prevalent [23]. The most prevalent cell type in the stroma of BC, CAFs, produce an abundance of chemokines, growth hormones, and ECM proteins that may encourage metastasis and dissemination [24]. Compared to their regular counterparts, the growth of tumors in xenograft models was greatly enhanced by fibroblasts from primary human invasive breast carcinomas [25]. The interaction of integrin three and CAF-derived interleukin 32 (IL32) at the tumor cell membrane, which enables the cross-talk between CAFs and breast tumor cells, promotes BC invasion [26]. GREMLIN 1, an antagonist of bone morphogenetic protein (BMP) signaling significantly impacts the connection between BC cells and CAFs, which encourages cancer cell invasion [27]. Another study claimed that BC’s epithelial-mesenchymal transition (EMT), invasion, and metastasis are caused by the exosome-derived microRNA-18b (miR-18b) from CAFs, which targets transcription elongation factor A like 7 (TCEAL7) and activates the nuclear factor-kappa B (NF-κB) pathway [28]. Also, through transfer from nearby CAF cells, patient-derived CAF exosomal miR-500a-5p can give BC cells an aggressive phenotype [29]. Given that CAF is frequently seen in brain metastases and that they encourage BC cell invasion, colony formation, and transmigration *in vitro*, they may also substantially impact the development of brain metastases in BC patients [30]. Furthermore, it has been demonstrated that signal transducer and activator of transcription 3 (STAT3) promotes the production of angiopoetin-like 4 (ANGPTL4), matrix metalloproteinase 13 (MMP13), and stanniocalcin-1 (STC1) by fibroblasts associated with the disease, which in turn promotes the growth of BC [31].

The triple-negative and HER2-positive BC subtypes have shown prognostic and predictive utility in immune cells and tumor-infiltrating (TI) lymphocytes (TILs) [32, 33]. T cells comprising cluster of differentiation 4^+^ (CD4^+^) helper cells, Forkhead box P3^+^ (FOXP3^+^), regulatory T (Treg), and effector cells such as NK cells and CD8^+^ T cells comprise most of the lymphocytes that infiltrate tumors [34]. TI Tregs interact with ECM elements, tumor and stromal cells, and other cells in the TME to generate an immune-suppressive phenotype. Due to Treg infiltration into the TME, TNBC is incredibly susceptible to medication resistance and early recurrence (TME) [35]. According to theory, the tumor creates an immunosuppressive microenvironment by secreting IL10 and transforming growth factor-β1 (TGF-β1) and increasing the activities of effector cells by releasing prostaglandin E2 and TGF-β1 signaling [36]. In the stroma of strongly perfused samples, close to but not inside blood vessels, most BCs are thought to have more CD4^+^ naive T cells than neighboring normal tissue. Their quantity is also related to TI Tregs, their clonotypic T cell receptor (TCR) sequences are most comparable to those of TI Tregs, and the presence of naive CD4^+^ T cells in tumors without CD8^+^ T cells is a blatantly unfavorable prognostic indicator.

Furthermore, it is known that TAM-derived C-C motif ligand 18 (CCL18) attracts the bulk of naive peripheral blood (PB) CD4^+^ T cells that differentiate into Tregs in human BC [37]. Though it is unknown how exosomes impact the environment around tumors, the relationship between exosomes and the immune system is vital in initiating and developing malignancies [38]. IL20 receptor subunit alpha (IL20RA) signaling, which explicitly enhances BC stemness and creates an immune milieu favorable to tumor growth, may be involved in forming numerous tumor formations [39]. Due to the tumor cells’ exceptional flexibility in response to extra-tumoral and various metabolic stimuli, the TME is essential [40].

TAMs, a significant cell population in BC, engage in a particular function that promotes tissue remodeling, angiogenesis, and suppression of adaptive immunity. Additionally, they produce a variety of compounds that aid in the development of tumors, including vascular endothelial growth factor (VEGF), cytokines, and enzymes that encourage angiogenesis, invasion, and metastasis [41]. According to recent research, epigenetically controlled genes mediate the communication between cancer and non-cancer host cells in the TME.

## Metastasis suppressor genes

The phrase “metastasis suppressor genes” refers to a class of genes that inhibits the ability of cancer cells to spread by concentrating on a critical stage of the invasion process without affecting tumorigenicity. To be precise, metastasis suppressors mainly influence downstream events other than basic tumor formation, whereas tumor suppressors inhibit metastasis by inhibiting primary tumor growth. The first metastasis suppressor, NME/Nm23 nucleoside diphosphate kinase (NDPK) 1 (NME1), was identified in 1988 by comparing the differentially expressed genes in high- and low-metastatic murine melanoma cell lines. Nm23 and Nm23-H1 are other names for NME1 [42]. More than 30 metastasis suppressors have been discovered based on the functional criteria. Both inside the cells and in the extracellular environment, metastasis suppressors have a variety of methods of action, and each one controls a different stage of the metastatic cascade [16, 43]. Not all the metastasis suppressor mechanisms are studied. However, some research suggests that it modulates the TME in various prospects, a potential therapeutic target for treating tumors. This review mainly focuses on some of the metastasis suppressor genes that suppress metastasis either inhibiting migration or colonization. This also includes the primary research of the genes in BC in particular *in vitro* studies. The activity of the metastasis suppressor gene in different BC cell lines and its effect on the TME is listed in Table 1.

**Table 1:** Metastasis suppressor genes and modulation of TME in BC cell lines

**Metastasis** **suppressor** **genes**	**Cell lines**	**Effect on the TME**	**References**
*BRMS1*	MDA-231 and MDA-435	Reduced localization of β1 integrin to adhesion-associated cellular protrusions	[44]
*KISS1*	MDA-MB-31Br and CN34Brm2Ctgl	Overexpression of epithelial marker E-cadherin	[45]
*NM23*	MDA-MB-435	Suppresses MT1-MMP activity	[46]
*GSN*	MCF and MDA-MB-231	TGF-β1 signaling leads to epigenetic alterations in gelsolin expression	[47]
*CADM1*	MCF7	EMT-induced E-cadherin and CADM1 modifications result in enhanced vulnerability to NK cell cytotoxicity	[48]
*KAI1*	MDA-MB-231	Induces apoptosis by the production of intracellular ROS and downregulates EGFR signaling	[49, 50]
*NDRG1*	MCF7 and MDA-MB-435	Upregulates E-cadherin and β-catenin by inhibiting SMAD/PSMAD32 and downregulation of TWIST, SNAIL, and SLUG	[51, 52]
*RhoGDI2*	MDA-MB-231	D4-GDI knockdown inhibits cell growth and invasion through activating Rac-dependent p38 and JNK signaling	[53]
*MAPK14*	MDA-MB-231, Hs587T, and SUM159	Cell migration, induction of EMT, p38 activation, and genomic regulation of NR4A1 expression by *cis*-acting-catenin/TCF/LEF complexes are all TGF-induced responses	[54]
*GAS1*	MDA	Decreases endothelial migration leading to decreased vascularization	[55]

*BRMS1*: BC metastasis suppressor 1; *GSN*: gelsolin; *CADM1*: cell adhesion molecule 1; *KAI1*: kangai1-anticancer; *NDRG1*: N-myc downstream-regulated gene 1; *RhoGDI2*: Rho guanine diphosphate-dissociation inhibitor 2; *MAPK14*: mitogen-activated protein kinase 14; *GAS1*: growth arrest specific 1; MT1-MMP: membrane type 1 MMP; ROS: reactive oxygen species; EGFR: epidermal growth factor receptor; PSMAD32: SMAD family member; TWIST: Twist family BHLH transcription factor; SNAIL: Snail family transcriptional repressor; D4-GDI: D4-guanine diphosphate-dissociation inhibitor; JNK: c-Jun N-terminal kinase; NR4A1: nuclear receptor subfamily 4 group A member 1; TCF: T cell factor; LEF: lymphoid enhancer factor

## BRMS1

Human BC and melanoma cell lines’ ability to metastasize is inhibited but not their tumorigenicity by the metastasis suppressor gene known as BRMS1, which is located at chromosome 11q13. This gene produces a protein that belongs to the mammalian switch independent 3 (mSin3a) family of histone deacetylase complexes (HDACs) and is primarily present in the nucleus. Hence it is convincible that the BRMS1 acts as a metastasis suppressor through epigenetic regulation [56]. BRMS1 suppresses metastatic regulation of BC subtypes, including TNBC, ER/PR^+^, ER^–^, PR^+^, HER2^–^, and HER2^+^ [57–61]. Since tumor promotion and prevention are delicately balanced by the immune system’s involvement, cellular interactions with the immune system may be impacted by BRMS1 [62, 63]. In the systemic circulation of immunocompromised mice, BRMS1 expression has been shown to drastically diminish the survival of BC cells [64, 65]. The expression of BRMS1 was assessed in cell lines such as MDA-MB-231, MDA-MB-435, MCF-10A, and HCC-1937.

BRMS1 was shown to be hypermethylated in MDA-MB-231, moderately methylated in HCC-1937 and MDA-MB-435, but unmethylated in MCF-10A when the DNA methylation status of BC cell lines, BC tissues, and associated non-malignant breast tissues was evaluated using major sperm protein (MSP) [66]. BRMS1 induces anoikis by upregulating the expression of pro-apoptotic genes that are associated with cells that are unable to adhere [65]. Cell adherence to the matrix is a crucial phase in determining whether the metastatic cascade is successful or unsuccessful at each stage. The remodeling of the cytoskeleton brought on by cell/matrix interaction is inhibited by BRMS1 expression. In BRMS1-expressing cells, adhesion-associated reductions in myocardin-related transcription factor (MRTF) and serum response factor (SRF) levels occur upon contact with the ECM; both proteins are intricately linked to the dynamic switching of globular (destabilized) to fibrillar (stabilized) actin filaments [67]. The oncogene Cullin3 encourages BC cells’ metastasis and EMT by degrading BRMS1 [68]. By reducing TWIST1 and SNAIL levels, BRMS1 significantly decreased TGF-β1-induced BC cells’ EMT and invasion [69]. JARID1C, a histone demethylase that stimulates the growth of cancer cells and controls transcription and chromatin remodeling, is overexpressed in BC, especially in cases where the disease has spread to distant organs. Increased migration and invasion result from JARID1C modification, suppressing BRMS1 expression by demethylating histone H3 lysine 4 (H3K4) and lowering H3K4me3 at the *BRMS1* gene promoter [70]. BRMS1 expression may serve as both a potential biomarker for selecting patients for clinical trials as well as a clinical prognostic indicator, capable of predicting relapse or response to targeted therapy. Recent research reveals that inhibiting the tumor necrosis factor (TNF)-related apoptosis-inducing ligand (TRAIL) pathway makes cells more susceptible to cell death, suggesting that BRMS1^+^ BC patients may benefit from TRAIL-targeted therapy [71].

## KISS1

KISS1 was first identified as a metastasis suppressor, but more recently, it has been found to play various roles in social behavior, reproduction, metabolism, and fertility [72]. It was given the name KISS1 in remembrance of the area where it was discovered—the home of Hershey’s chocolate “Kisses” [73]. In 1996, when human chromosome 6 was introduced to a cancer cell, the lab of Dr. Danny Welch in Hershey, Pennsylvania, isolated a complementary DNA (cDNA) from the cell, preventing metastasis [74]. It can inhibit melanoma and BC metastasis. The KISS1-derived peptides were given the kisspeptins (KPs)—KP54, 14, 13, and 10—following the number of amino acids in the peptide chain. Due to its anti-metastatic properties, one of them, KP54, was initially known as metastin [75]. When blocking KISS1 with a specific short peptide antagonist (p234) impacts TGF-mediated cell invasion and MMP9 synthesis, it is proven that KISS1 plays a vital role in mediating the pro-invasive effects of TGF as a downstream target of the canonical TGF/SMAD2 signaling pathway [76]. MMP9 and other proteases encourage tumor invasion by destroying the ECM. The tumor-suppressive role is thought to prevent tumor invasion by suppressing MMP9 production and activity and MAPK [77]. Maintaining the epithelial state and reducing BC cell invasiveness are two effects of KISS1 signaling through PKD1, which also performs tasks related to its function as a metastasis suppressor. In TNBC cells, melatonin boosted the expression of KISS1, which was mediated through GATA binding protein 3 (GATA3). Melatonin’s inhibition of KISS1 remained as a result [78]. IL30 increased the proliferation, motility, and inflammatory environment in TNBC cells, aiding KISS1-dependent metastasis. Treatment with IL30 *in vivo* enhanced the expansion of intra-tumoral CD11b^+^/Gr1^+^ myeloid cell infiltrates, vascular dissemination, and cancer cell proliferation in TNBC [79]. It was demonstrated that the KISS1 receptor (KISS1R) regulated the expression of the drug efflux transporter BC resistance protein (BCRP), a crucial regulator of the multi-drug resistance phenotype in BC significantly elevated in TNBC, demonstrating that KISS1R-induced drug resistance was dependent on the activity of adenosine triphosphate-binding cassette (ABC) transporters [80]. KISS1 stops the migration of BC cells by inhibiting the NF-κB pathway and RhoA activation that TNF-α causes. Although KP10 did not impact cancer cell proliferation, KISS1 overexpression and KP10 stimulation reduced TNF-α-induced NF-κB activity and suppressed TNF-α-induced cell migration, respectively, and lowered TNF-α-induced cell attachment to fibronectin in BC cells [81]. According to studies on the function of KISS1 in MDA-MB-231 and MDA-MB-157 cells, the suppression of MMP9 and MMP2 activity is linked to the reduction of metastasis caused by KP [82]. NF-κB p50 and MMP9 expression were discovered to be adversely linked with the expression of the KISS1-1 protein [83]. According to some studies, the stromal cell-derived factor-1 (SDF-1)/C-X-C chemokine receptor type 4 (CXCR4) system may be crucial for BC invasion and EMT. KP-10 therapy inhibits CXCR4 expression to decrease invasion, and EMT brought on by SDF-1 [84]. A recent study found that TGF-β1, but not SMAD, inhibits human trophoblast cell invasion by increasing KP synthesis through the extracellular signal-regulated kinase 1/2 (ERK1/2) signaling pathway [85].

## Non-metastatic 23

The non-metastatic (Nm) protein 23 H1 is the molecular name for the common enzyme (NDPK A, Nm23-H1). Steeg et al. [42, 86] initially discovered this metastasis suppressor protein. The human *Nnm23* genes Nm23-H1 and Nm23-H2 have been located on chromosome 17q21. The phosphorylation of Nm23/NME histidine 118, which is implicated in the activities of the enzymes NDPK and histidine protein kinase (HPK), has received considerable attention. The processes through which Nm23 inhibits metastasis and motility still need to be better understood [87]. Loss of heterozygosity, spontaneous mutations, and polymorphisms in the *Nm23* gene are rarely found in malignancies; as a result, Nm23 protein levels are likely to impact the tumor cells’ ability to metastasize [88]. Previously, in the human MDA-MB-435 (BC) and K-1735 TK (melanoma) cell lines, the overexpression of Nm23 was linked to a reduction in tumor-spreading potential [89]. EMT, triggered by stress caused by tumor hypoxia and growth factor depletion, leads to invasion and metastasis and is linked to CSCs characteristics. In Panc-1/MDA-MB-231 cells, serum starvation and hypoxia reduced the expression of the prototypical Nm23-H1 [90]. In past investigations, the less aggressive MCF-7 cells, which express much of Nm23-H1, and the invasive MDA-MB-231 cells, which express little to no Nm23-H1, demonstrated a relationship between messenger RNA (mRNA) levels and protein expression. This implies that transcriptional processes might control the expression of Nm23-H1. Several transcription factors, including CCCTC-binding factor (CTCF) and early growth response factor 1 (EGR1), can influence the transcription of NME1. This might either result in BC cells expressing more Nm23-H1, which would promote a phenotype that is less likely to spread, or it could result in Nm23-H1 production being reduced [91]. Treatment with an NDPK A and B inhibitor or a purinoreceptor antagonist attenuates the effects of MDA-MB-231 extra vesicles on the mice’s pulmonary vascular leakage and experimental lung metastases [92]. An additional experiment demonstrates that NME1 (Nm23-H1) was found to be the primary component of these exosomes when it was transfected into exosomes formed from two cancer cell lines (MDA-MB-231T and MDA-MB-435). These exosomes altered the endocytic pathways of receiving tumor cells via NME1, decreasing their motility and migration *in vitro* more than exosomes from control transfectants [93]. Invasive breast carcinomatous lesions exhibit a highly anti-correlated expression of NME1 and cortical MT1-MMP, according to RNA sequencing (RNAseq) data [46].

## GSN

*GSN* is the first tumor suppressor gene in human basal carcinoma to be impacted by histone acetylation [94]. The actin-binding protein GSN plays a crucial role in controlling the formation and disassembly of actin filaments. Additionally, it was revealed that specific tumor cells had lower cellular levels of GSN and that overexpressing GSN by gene transfer reduces tumorigenicity. GSN’s carboxyl terminus is crucial for suppressing metastasis, reducing chemotaxis, and delaying cell spreading [95]. In MCF-7 cells, down-regulating GSN resulted in a concentration-dependent increase in migratory activity and *vice versa* [96]. GSN’s epigenetic alteration by TGF-β1 may affect the EMT process in BC cells [47]. Results from gel shift and supershift assays, Southwestern blotting studies, and gel shift and supershift assays suggest that the 27-bp GSN *cis*-element is preferentially bindable by activating transcription factor 1 (ATF1) in cancer cells. This shows that ATF1 is involved in silencing the GSN promoter, as opposed to its transactivating effect on other types of promoters [97]. The study suggests that GSN is downregulated through epigenetic processes.

Furthermore, the more significant role in this repression is played by hypoacetylation of histones as opposed to hypermethylation of regulatory cytosine-guanine (CpG) sites [94]. Rodent and human BCs share a common dysregulation of cyclin D1 and GSN, and it appears that, at least in part, a changed transcription rate is responsible for both genes’ malfunction [98]. On the other hand, several studies assert that the invasive motile characteristics of cells and cell aggregation are considerably diminished in various cancer cell types, including MDA-MB-231 and PC-3 cells when CapG or GSN is downregulated. These findings suggest that GSN and CapG may operate as tumor activators [99]. Thus, further research needs to be done to understand the role of GSN clearly.

## CADM1

CADM1 or Tslc1, Necl2, Ra175, IgSF4a, and SynCAM, a transmembrane protein member of the immunoglobulin superfamily is predominantly implicated in cell-cell interactions [100]. TSLC1 is highlighted as a tumor suppressor in lung adenocarcinoma [101], but recent studies have highlighted the metastatic suppressive properties of CADM1 in breast carcinomas [102].

From a macro-perspective, higher clinical presentations of CADM1 have been correlated with tumor size and staging [103, 104]. Studies have co-related poor patient survival rates due to hypermethylation of the promoter region of the *CADM1* gene leading to transcriptional repression in metastatic BC. At the same time, very little research has gone into understanding the molecular aspects of its mechanisms [105–108]. Research studies have implicated CADM1’s protective role in several stages of the metastatic cascade in BC [109] and ovarian cancer [110].

A study conducted by Faraji et al. [102] explored the anti-metastatic and anti-proliferative roles of CADM1 in association with components involved in immunosurveillance in the TME. Expression of CADM1 in metastatic murine mammary cell lines suppressed metastasis without affecting primary tumorigenesis. At the same time, the scratch wound assay demonstrated a significant reduction in the relative motility of CADM1^+^ BC cells compared to the control specimen [102]. Furthermore, when CD8^+^ T cells were reduced in immune-competent mice, the metastasis-suppressing impact of CADM1 was partially phenocopied in mice lacking T cell-mediated immunity [102].

Cancer development and progression are impacted by cell cytotoxicity and tumor rejection caused by CD8^+^ T cell-mediated immunity and NK cells [111]. Class-I restricted T cell adhesion molecule (Crtam) has been shown to interact with CADM1 and regulate immunoediting processes by generating interferon, which may stimulate pro-inflammatory signals to induce cytotoxicity. It is a crucial biomarker of activated CD8^+^ T cells and NK cells [112, 113]. The hypothesis was tested by Faraji et al. [102] and team and a significant increase in interferon-γ levels from the lymph nodes of mice comprising cadm1 expressing neoplasms was observed. Reports also show NK-mediated cytotoxicity depends on the balance of cellular E-cadherin (a noted marker for EMT) and CADM1 owing to homophilic interactions mediated by membrane-associated guanylate kinases and CADM1’s C-terminal intracellular domain [114, 115].

CADM11 has much potential to be targeted for therapeutic applications in invasive late-stage cancers owing to the co-relation between non-coding RNA expression and CADM1 concentration. Several reports indicate the down-regulation of CADM1 due to the expression of long non-coding (lncRNA) and miR. A study conducted by Zhang et al. [103] conclusively demonstrated the effects of miR-155-3p associated with the downregulation of CADM1, leading to tumor progression and metastasis, and apoptosis. Hence, CADM1 looks to be a promising and potential target to treat multiple late-stage metastatic cancers.

## CD82

KAI1/CD82 was first identified in T-cell activation research and is a type III transmembrane glycoprotein member of the tetraspanin protein family. It is also known as R2, C33, IA4, or 4F9 [116, 117]. Studies identified the *KAI1* gene on human chromosome 11, which comprises ten exons and nine introns [118]. Tetraspanins are proteins on the cell membrane consisting of 4 putative domains, N-terminal and C-terminal, and small and large extracellular domains. Overall functional activity of the KAI1 protein is influenced by its domains where glycosylation of its N-terminal is crucial for adhesion molecules and cell surface receptors. At the same time, polar molecules spanning the cellular membrane are associated with the maintenance of protein conformation and cell migration, invasion, and metastasis [119–121].

The metastatic cascade involves various steps such as intravasation, cell migration, extravasation, and colonization [122]. KAI1 protein is known to down-regulate intercellular adhesion processes and prevent the colonization of metastatic cancer cells [123]. The alteration of cell adhesion factors as such in the TME regulates the migratory behavior and metastatic potential of cancer cells. Overexpression of KAI1/CD82 induces apoptosis via the production of intracellular ROS, which is thought to be the downstream product of the release of intracellular anti-oxidant glutathione [124].

*In vitro*, studies that established CD82’s anti-metastatic function specifically in mammary tumor cells and found that knocking down CD82 in highly metastatic MDA-MB-231 BC cells promoted functional inactivation of MAPK pathway and EGFR signaling, which increased cell migration and invasiveness, were found to be correlated with the metastatic suppressive effects of KAI1/CD82 [49, 50]. Studies by He et al. [125] and Odintsova et al. [126] reveal that CD82 slows the downregulation of EGFR signaling in the tetraspanin web which describes the lateral interactions between tetraspanins and their partners in the cell membrane and is crucial for developing tumor cells. Multiple reports indicate KAI1/CD82 interacts with tetraspanin protein molecules such as: (A) CD9 and CD81 [127, 128]; (B) β1 and β2 integrins that lead to the formation of p130CAS–Crk complex, thus regulating inhibition of cell migration [129–133]; (C) major histocompatibility complex (MHC) class I and II molecules for immunomodulation in the TME [133, 134]; (D) heparin-binding epidermal growth factor [135] desensitizes epidermal growth factor signaling via endocytosis of EGFR to suppress metastasis [126]; (E) intracellular signaling molecules such as protein kinase C recruit the integrin-tetraspanin protein complex to regulate cell motility and migration [136] and are also connected to the urokinase-type plasminogen activator receptor (uPAR)’s intracellular dispersion into focal adhesions, which is mediated by the α5β1 integrins [137].

## NDRG1

NDRG1 is a member of the 4 *NDRG* gene family determined to suppress oncogenesis and tumor progression in different cancers such as breast, prostate, brain, colon, pancreas, and rectum [138, 139]. Contradictory evidence also suggests the oncogenic effects of NDRG1 in kidney and liver cancers [138].

The mature protein is said to be primarily located in the cytoplasm but is also dependent on cell type; for instance, NDRG1 is predominantly found in the plasma membrane of lactating mammary epithelial cells, the inner membrane of the mitochondria in cells lining the proximal renal tubule and nucleus of prostatic epithelial cells [138, 139]. NDRG1 has been to said disrupt several signaling pathways as a pivotal response to regulate cell proliferation, cell-cell adhesion, and motility under stress conditions like hypoxia and reoxygenation and also have varying effects on the TME [138, 140–142].

Several chemical and biological signals regulate NDRG1 concentration in the cell [140]. Epigenetic modifications to the *NDRG1* gene can lead to reduced expression. In contrast, several factors can increase expressions, such as heavy metal ions, iron depletion, hypoxia-inducible factor 1, and DNA-damaging agents [138, 139, 143]. It is also said to decrease the expression of p53 under hypoxic conditions, thus preventing apoptosis [144]. Various reports have linked the expression of NDRG1 with suppressing tumor growth and development [51, 52, 145]. It mediates the upregulation of several epithelial markers like E-cadherin and β-catenin and downregulates mesenchymal phenotypes occurring in EMT via inhibiting SMAD/PSMAD32 expression and downregulating SNAIL, SLUG, and TWIST expression to suppress cell migration and metastasis [51, 52]. NDRG1 expression in cancer cells is also associated with inhibition in β-catenin phosphorylation at Ser33/37 and Thr41 residues which protect the cell from β-catenin, a widely known pro-oncogenic transcription factor in the Wnt signaling pathway and thus suppresses cancer metastasis [52, 146]. This statement is backed up by a study that showed suppressed metastasis in mouse mammary cancer models [147]. Reports also indicated decreased expression of ErbB receptors such as EGFR, HER2, and HER3 to NDRG1 expression preventing the formation of HER2/HER3 dimers and disrupting major oncogenic pathways primarily associated with mammary tumors [145, 148]. Stabilizing the RhoGDI-cell division control (CDC42) complex, which produces cell protuberances at the cell’s leading edge in colorectal cancer cell lines, is another mechanism through which NDRG1 is connected to increased invasiveness [142]. Hence, metastatic suppressive activities at the molecular level are observed with NDRG1 expression and can be further explored.

**Figure 2:**
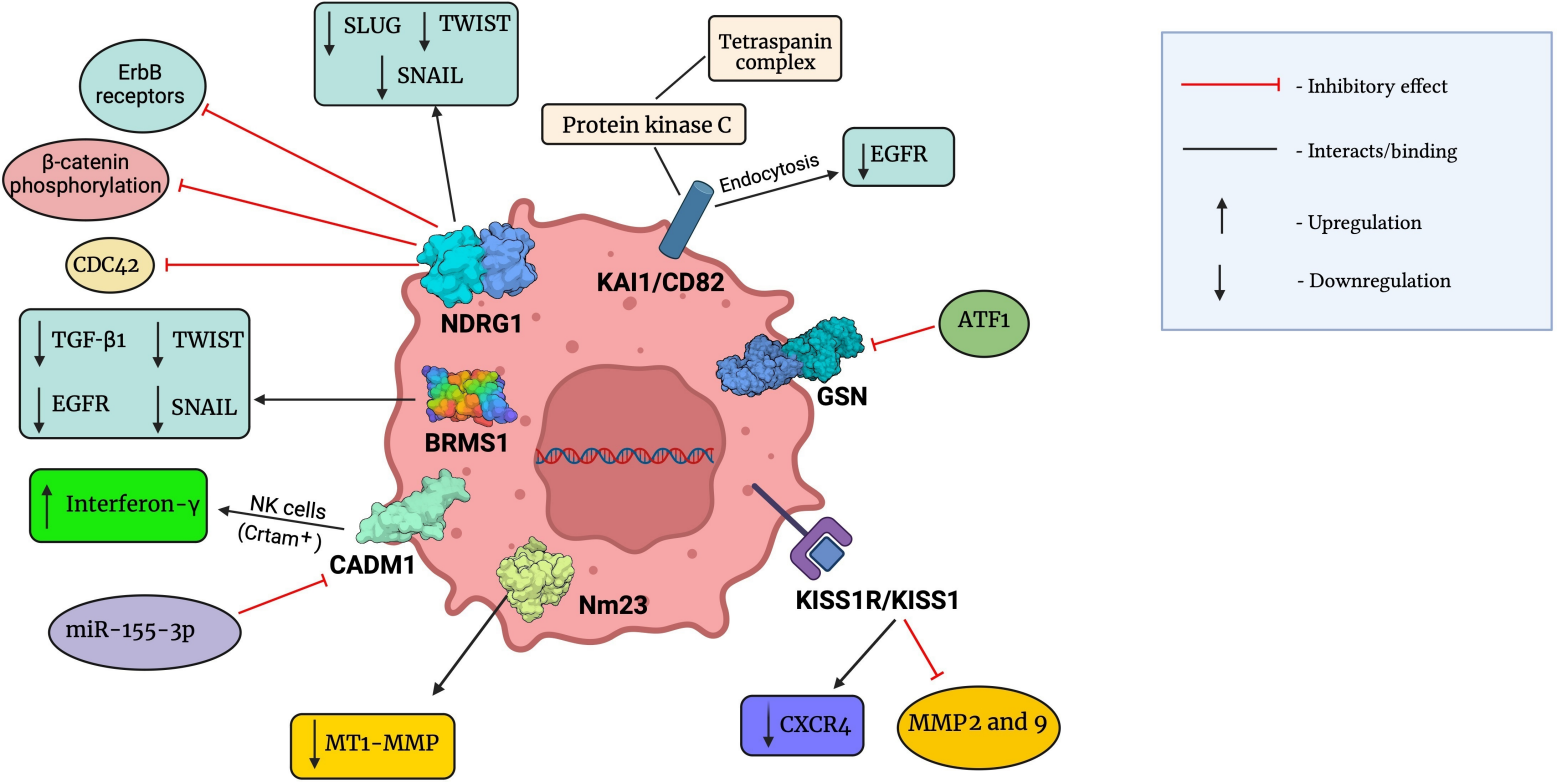
Molecular interactions between metastatic suppressors and chemical components of the TME. This figure was created with BioRender

However, overwhelming contradictory evidence links the expression of NDRG1 with oncogenic effects in aggressive BCs [149–152]. A study correlated increased expression of NDRG1 with advanced tumor progression, thus highlighting it as an important prognostic biomarker in invasive BC [152]. Hence, NDRG1 needs to be further explored and studied to develop into a novel therapeutic agent against cancer. A summary of molecular interaction between these metastatic suppressors and chemical components of the TME has been given in Figure 2.

## Conclusions

Due to a greater understanding of the molecular process behind the initiation and progression of cancer, targeted cancer therapies have achieved tremendous advancements in recent decades [153]. Many biological functions in the stage of metastasis are controlled by metastasis suppressor genes [154]. An increased chance of abnormal cell proliferation, differentiation, cell death, and cancer development exists when metastasis suppressor genes are not functioning properly. The downregulation of metastasis suppressor genes has been discovered in various cancers like breast, pancreatic, ovarian, bladder, colorectal, neck, uterine, and lung cancer. One of the reasons for this downregulation can be the modulation in the TME such as lymphocytes, CAF, TAM, ECM, exosomes, CTCs, etc. New delivery techniques and more sophisticated gene expression systems are being studied as potential cancer treatments and cures. Lack of tumor selectivity is one of the problems with current cancer treatments. Therefore, one of the main goals of cancer gene therapy is to specifically target tumor cell gene expression [155]. The classification of the BC subtypes, specifically HR^+^/HER2^+^, HER2^+^, and HR^+^/HER2^–^, determines the majority of treatment plans. Local treatments for Nm BCs include surgery (which may need axillary lymph node removal) and radiotherapy [156]. Different directed gene therapy strategies have been created employing tumor-specific promoters to treat BC [157]. After screening a library of recombinant secreted microenvironmental proteins, fibroblast growth factor 2 (FGF2) was discovered to be an essential regulator of anti-estrogen resistance, mechanistic target of rapamycin complex 1 (mTORC1) inhibition, and phosphatidylinositol 3-kinase inhibition in ER^+^ BC [158]. When used in conjunction with oncolytic virotherapy for the treatment of breast and other metastatic cancers, KISS1 transgenic expression has the potential to be an efficient tool for CRAd-mediated cytotoxicity in BC cells [159]. Additionally, it is true that customized care and precision medicine, which are directed at specific patients and patient subgroups, are gaining popularity in oncology, where the focus is on delaying the onset of the disease and the treatment plan is devised to maximize effectiveness while minimizing side effects [160]. lncRNA-based therapy and miR therapeutics are the other treatments for BC based on epigenetic drugs by which metastasis suppressor genes are epigenetically inactivated [156]. Further studies about the TME and the metastasis suppressor genes need to be done to recognize their exact role in tumors. The TME contributes to the growth and invasion of cancer cells and their components help the cells for migration. When suppressor genes enter the scene to inhibit the metastatic process of tumor cells, what role do the components of the TME play? Will the interaction of these components and genes make any remarkable changes in the metastatic cascade? If so, then how can this be used as a potential target for therapies? To answer these questions, a detailed review has been done to study the relationship between the TME and the metastasis suppressor genes in BC. Some of the metastasis suppressor genes are downregulated which leads to metastasis in several tumors. Some studies suggest that modulation in the TME is associated with cancer progression and proliferation. Hence, understanding the role of each metastasis suppressor gene in the TME is important in order to of them as a potential therapeutic target.

## Abbreviations

ATF-1: activating transcription factor-1
BC: breast cancer
BRMS1: breast cancer metastasis suppressor 1
CADM1: cell adhesion molecule 1
CAFs: cancer-associated fibroblasts
CD4^+^: cluster of differentiation 4^+^
CXCR4: C-X-C chemokine receptor type 4
D4-GDI: D4-guanine diphosphate-dissociation inhibitor
ECM: extracellular matrix
EGFR: epidermal growth factor receptor
EMT: epithelial-mesenchymal transition
ERα: estrogen receptor alpha
GSN: gelsolin
HER2: human epidermal growth factor 2
IL32: interleukin 32
KAI1: kangai1-anticancer
KISS1R: KISS1 receptor
KPs: kisspeptins
MAPK: mitogen-activated protein kinase
miR-18b: microRNA-18b
MMP13: matrix metalloproteinase 13
MT1-MMP: membrane type 1 matrix metalloprotease
NDPK: nucleoside diphosphate kinase
NDRG1: N-myc downstream-regulated gene 1
NF-κB: nuclear factor-kappa B
NK: natural killer
Nm: non-metastatic
NME1: NME/Nm23 nucleoside diphosphate kinase 1
PR: progesterone receptor
SNAIL: Snail family transcriptional repressor
TAMs: tumor-associated macrophages
TGF-β1: transforming growth factor-β1
TME: tumor microenvironment
TNBC: triple-negative breast cancer
TNF: tumor necrosis factor
Treg: regulatory T
TWIST: Twist family BHLH transcription factor

## Declarations

### Copyright

© The Author(s) 2023.
